# Isotopic Values of Prenatal Development: *δ* ^13^C and *δ* ^15^N Variation in Early‐Formed Human Tissues

**DOI:** 10.1002/ajpa.70251

**Published:** 2026-04-20

**Authors:** Tamara Leskovar, Doris Potočnik, Marjeta Mencin, Nives Ogrinc, Christophe Snoeck, Matija Črešnar

**Affiliations:** ^1^ Faculty of Arts, Department of Archaeology, Centre for Interdisciplinary Research in Archaeology University of Ljubljana Ljubljana Slovenia; ^2^ Department of Environmental Sciences Jožef Stefan Institute Ljubljana Slovenia; ^3^ Archaeology, Environmental Changes and Geo‐Chemistry Vrije Universiteit Brussel Brussels Belgium

**Keywords:** *δ* ^13^C and *δ* ^15^N, different skeletal tissues, early life physiology, perinatal/neonatal/infant remains, stable isotopes

## Abstract

**Objectives:**

Carbon (*δ* ^13^C) and nitrogen (*δ* ^15^N) isotope analysis illuminates diet and physiology in the past, yet interpretation is complicated by development and tissue‐specific collagen formation. This study tests whether systematic isotopic offsets occur between auditory ossicles, deciduous dentin, long bones, and ribs in individuals who died as perinate, neonate, or young infants, and assesses implications for reconstructing maternal diet and early‐life physiology.

**Materials and Methods:**

Collagen *δ* ^13^C and *δ* ^15^N were measured for 106 samples from 27 individuals recovered from five sites spanning the Early/Late Iron Age, Early Roman, and 17th–19th centuries.

**Results:**

Results reveal a consistent *δ* ^15^N trajectory: values generally increase from ossicles to teeth and decrease from teeth to long bone/rib. Tooth–long bone/rib *δ* ^15^N offsets are significant across the sample and within each age group. *δ* ^13^C shows a modest, systematic increase from ossicle to tooth; ossicles are significantly lower than long bones/ribs and tooth, whereas tooth to long bone/rib *δ* ^13^C exhibits no uniform group‐level offset. These systematic patterns cannot be explained by maternal dietary variation across time and sites and instead reflect developmental physiology.

**Discussion:**

These findings indicate that, in perinates, neonates, and young infants (> 4.5 months old), physiological conditions associated with different developmental stages and tissue turnover rather than diet govern collagen isotope variation. Ossicles predominantly archive mid prenatal physiology; deciduous dentin emphasizes late gestation (±immediate perinatal); long bone/rib integrates and dampens signals over a broader interval. This study recommends tissue‐aware sampling and reporting when inferring maternal diet, fetal physiology, and early‐life stress from collagen.

## 1 Introduction

Carbon (*δ* ^13^C) and nitrogen (*δ* ^15^N) isotope analysis in human skeletal remains have contributed to the understanding of past populations and individuals. It can provide data needed to explore past human lives and societies, such as dietary habits and weaning, trophic positions, and physiological stress (Fuller et al. 2006; Katzenberg and Waters‐Rist 2018; Mekota et al. 2006). In subadult individuals–particularly infants and perinates–these isotopic values reflect not only dietary intake but also physiological status and maternal input in utero and during early life (Beaumont et al. 2015; Prowse et al. 2008; Tsutaya and Yoneda 2013).

The interpretation of isotopic data in the youngest individuals is especially complex due to differences in developmental timing and tissue metabolism. Perinatal and neonatal individuals—those who died in utero (typically third trimester), during birth, or within the first weeks of life—offer a unique opportunity to examine exclusively prenatal or early postnatal isotopic signals.

Comparative studies have highlighted consistent differences between dentin and bone collagen in older subadults and adults (Beaumont et al. 2013, 2018; Burt 2013, 2015; Haydock et al. 2013), often attributed to the different time periods represented by these tissues. Tooth dentin, once formed, does not remodel and thus preserves a time‐specific isotopic record, while bone remodels continuously and averages dietary signals over weeks to years (Hedges et al. 2007; Hillson 2014). Interestingly, isotopic differences have also been observed in individuals whose dentin and bone were forming concurrently, such as neonates, suggesting a physiological rather than purely temporal cause (Beaumont et al. 2018).

These discrepancies may be influenced by tissue‐specific differences in protein metabolism and amino acid routing. Previous research suggests that *δ* ^15^N values in fetal tissues may be slightly elevated due to nitrogen recycling and heightened protein retention efficiency (Fuller et al. 2006; Tsutaya and Yoneda 2013), while *δ* ^13^C values appear to be more stable and primarily reflect maternal dietary input (O'Connell and Hedges 1999; Tsutaya and Yoneda 2013). However, subtle *δ* ^13^C offsets between tissues may also occur due to differences in collagen synthesis pathways (Fuller et al. 2005a; O'Connell and Hedges 1999).

Despite these findings, few studies have systematically compared *δ* ^13^C and *δ* ^15^N isotopic composition in multiple skeletal tissues (e.g., tooth, long bone, rib, and ossicle) in individuals forming these tissues exclusively in utero or shortly after birth. One rare case of a neonate from Raunds Furnell (Beaumont et al. 2018) revealed significant differences between rib and dentin collagen. Burt (2015), however, found no such difference in a limited number of fetal individuals, including perinates, neonates and infants, from Fishergate House.

Tooth and bone development proceed along distinct biological pathways. Tooth development is tightly programmed, with deciduous incisors and molars initiating formation from the second trimester onward (30 weeks in utero) and progressing into infancy (AlQahtani et al. 2010; Hillson 2014). Bone forms either through intramembranous ossification (e.g., skull vault) or endochondral ossification (e.g., long bones, ribs), both beginning early in gestation (Clarke 2008; Galea et al. 2020; Ortega et al. 2004). Long bones and ribs begin ossifying between 7 and 9 weeks of gestation and continue to remodel throughout fetal life and infancy (Blumer 2021; Scheuer and Black 2000). Auditory ossicles, tiny bones of the middle ear, begin ossifying with the incus at 14 weeks in utero, followed by malleus at 15–16 weeks and stapes at 18 weeks in utero. The ossification is almost completed by the 32–35 week in utero and ossicles achieve their final shape and dimensions at birth. Only the trabecular fascicle of malleus and incus and bone marrow cavities are finalized 25 months after birth. Although some minor remodeling may continue briefly after birth, ossicles are generally considered to preserve a fixed prenatal isotopic signal (Hanson and Anson 1962; Lasky and Williams 2005; Richard et al. 2017; Rolvien et al. 2018).

Given these differences, interpreting isotopic signals from perinatal remains requires careful attention to both developmental timing and physiological context. While *δ* ^15^N values in fetal tissues may be elevated due to nitrogen recycling and retention in the maternal‐fetal system (Fuller et al. 2006; Tsutaya and Yoneda 2013), *δ* ^13^C value is expected to show less metabolic distortion and more faithfully represent maternal diet. *δ* ^13^C is often less metabolically perturbed than *δ* ^15^N in infants (Fuller et al. 2006; Tsutaya and Yoneda 2013), yet tissue‐specific growth/turnover can still shift collagen *δ* ^13^C and *δ* ^15^N values (Beaumont et al. 2015, 2018; Eerkens et al. 2011).

This study reports carbon (*δ* ^13^C) and nitrogen (*δ* ^15^N) isotope values from tooth and bone collagen of perinatal and neonatal individuals (≈30–40 gestational weeks and shortly before/after birth) and early infants (≈1.5–4.5 months) from multiple archeological sites in Slovenia. The primary objective of this study is to test the hypothesis that systematic isotopic offsets occur between tissues formed at different stages of prenatal development, reflecting differences in tissue formation timing and associated physiological conditions rather than dietary input. It is expected that earlier‐forming and continuously remodeling tissues such as ribs and long bones will show isotopic values that integrate over a longer developmental interval and reflect composite physiological states during growth, whereas later‐forming tissues such as prenatal dentin will exhibit isotopic values more restricted to the specific physiological conditions present during their shorter and later formation window. In cases where infant material is included, postnatal tissues may show limited isotopic adjustment toward values reflecting early metabolic independence.

## 2 Materials and Methods

### 2.1 Samples

All the details on samples are provided in Table 1. Stable isotope values (*δ* ^13^C and *δ* ^15^N) were obtained from tooth dentin, long bone, rib and auditory ossicle collagen from perinatal, neonatal and infant individuals across five archeological sites in Slovenia, dating from Early (Congress Square; *n* = 1) and Late Iron Age (Škocjan; *n* = 2), early Roman period (Congress Square; *n* = 10), to 17th–19th century Modern period (Čemšenik; *n* = 1, Vrazov trg; *n* = 1, Polje; *n* = 12). Where preservation allowed, multiple tissues were analyzed for each individual. Sites representing different chronological periods were included primarily on the basis of sample preservation and availability of well‐documented perinatal and neonatal remains. The comparison focuses on development rather than cultural variation, as the physiological processes underlying fetal and early postnatal tissue formation are expected to be consistent through time.

**TABLE 1:** Sample list with the results of isotope analyses.

Site	Grave	Age	Period	Element	*δ*13Ccol (‰)	*δ*15Ncol (‰)	C/N
Congress Sq.	1000	40 w in utero ±2 w	Early Roman	m1	−12.57	12.39	3.5
Congress Sq.	1000	40 w in utero ±2 w	Early Roman	Rib	−12.03	11.31	3.3
Congress Sq.	1000	40 w in utero ±2 w	Early Roman	Femur	−12.5	11.28	3.6
Congress Sq.	1001	40 w in utero ±2 w	Early Roman	i2	−18.37	12.68	3.5
Congress Sq.	1001	40 w in utero ±2 w	Early Roman	Rib	−18.83	11.01	3
Congress Sq.	1001	40 w in utero ±2 w	Early Roman	Femur	−18.84	11.16	3.1
Congress Sq.	1052	34–36 w in utero	Early Roman	i1	−18.54	11.19	3.6
Congress Sq.	1052	34–36 w in utero	Early Roman	i2	−18.65	10.89	3.9
Congress Sq.	1052	34–36 w in utero	Early Roman	Rib	−19.01	9.94	3.3
Congress Sq.	1052	34–36 w in utero	Early Roman	Tibia	−18.89	9.98	3.4
Congress Sq.	1055	38–40 w in utero	Early Roman	m1	−19.01	12.09	3.3
Congress Sq.	1055	38–40 w in utero	Early Roman	m1	−19.02	12.28	3.4
Congress Sq.	1055	38–40 w in utero	Early Roman	Rib	−19.47	10.52	3.2
Congress Sq.	1055	38–40 w in utero	Early Roman	Femur	−19.27	10.56	3.6
Congress Sq.	1056	40 w in utero ±2 w	Early Iron Age	m1	−13.26	10.72	3.6
Congress Sq.	1056	40 w in utero ±2 w	Early Iron Age	Rib	−13.88	9.12	2.9
Congress Sq.	1056	40 w in utero ±2 w	Early Iron Age	Femur	−14.11	8.92	2.9
Congress Sq.	1058	30–34 w in utero	Early Roman	m1	−18.03	12.24	3
Congress Sq.	1058	30–34 w in utero	Early Roman	m1	−17.34	12.05	3.5
Congress Sq.	1058	30–34 w in utero	Early Roman	Rib	−18.12	10.9	3.5
Congress Sq.	1058	30–34 w in utero	Early Roman	Humerus	−18.05	10.8	3.5
Congress Sq.	1060	30 w in utero ±1 m	Early Roman	i1	−17.52	11.18	3.3
Congress Sq.	1060	30 w in utero ±1 m	Early Roman	Rib	−17.94	9.76	3
Congress Sq.	1060	30 w in utero ±1 m	Early Roman	Humerus	−17.84	9.67	3.2
Congress Sq.	1062	40 w in utero ±2 w	Early Roman	i1	−17.63	11.36	3.4
Congress Sq.	1062	40 w in utero ±2 w	Early Roman	m1	−17.18	11.48	3.5
Congress Sq.	1062	40 w in utero ±2 w	Early Roman	m2	−17.79	11.31	3.5
Congress Sq.	1062	40 w in utero ±2 w	Early Roman	Rib	−18.31	10.25	3.3
Congress Sq.	1064	38–40 w in utero	Early Roman	i1	−18.14	11.78	3.6
Congress Sq.	1064	38–40 w in utero	Early Roman	Rib	−18.46	10.51	3.3
Congress Sq.	1066	38–40 w in utero	Early Roman	i1	−18.51	11.84	3.4
Congress Sq.	1066	38–40 w in utero	Early Roman	Rib	−19.18	10.21	3.1
Congress Sq.	1066	38–40 w in utero	Early Roman	Femur	−19.17	10.12	3
Congress Sq.	1068	40 w in utero ±2 w	Early Roman	i1	−18.66	11.83	3.5
Congress Sq.	1068	40 w in utero ±2 w	Early Roman	m1	−18.73	11.54	3.4
Congress Sq.	1068	40 w in utero ±2 w	Early Roman	m2	−18.92	11.76	3.4
Congress Sq.	1068	40 w in utero ±2 w	Early Roman	Rib	−19.17	10.37	3.2
Congress Sq.	1068	40 w in utero ±2 w	Early Roman	Tibia	−19.2	10.38	3
Congress Sq.	1068	40 w in utero ±2 w	Early Roman	Ossicle	−19.04	10.91	3.4
Škocjan	1	30–40 w in utero	Late Iron Age	m1	−13.82	12.13	3
Škocjan	1	30–40 w in utero	Late Iron Age	m1	−14.06	12.17	3.3
Škocjan	1	30–40 w in utero	Late Iron Age	Rib	−14.28	10.47	3.4
Škocjan	1	30–40 w in utero	Late Iron Age	Femur	−14.25	10.46	3
Škocjan	1	30–40 w in utero	Late Iron Age	Ossicle	−14.64	10.91	3
Škocjan	2	40 w in utero ±2 w	Late Iron Age	m1	−13.71	12.3	3.4
Škocjan	2	40 w in utero ±2 w	Late Iron Age	m1	−13.72	12.39	3.5
Škocjan	2	40 w in utero ±2 w	Late Iron Age	Rib	−14.55	10.79	3.4
Škocjan	2	40 w in utero ±2 w	Late Iron Age	Humerus	−14.47	10.97	3.1
Čemšenik	30	40 w in utero ±2 w	Modern	i1	−17.74	9.56	3.3
Čemšenik	30	40 w in utero ±2 w	Modern	Rib	−17.49	8.53	3.2
Čemšenik	30	40 w in utero ±2 w	Modern	Ossicle	−17.24	8.2	3.3
Vrazov	3147	36–38 w in utero	Modern	m1	−22.31	15.93	3.4
Vrazov	3147	36–38 w in utero	Modern	Rib	−19.74	11.39	3.5
Vrazov	3147	36–38 w in utero	Modern	Femur	−19.79	11.36	3.5
Polje	156	30–36 w in utero	Modern	i1	−20.89	11.53	3.4
Polje	156	30–36 w in utero	Modern	i1	−21.36	12.22	3.4
Polje	156	30–36 w in utero	Modern	Rib	−19.82	10.36	3.3
Polje	156	30–36 w in utero	Modern	Metacarpal	−19.07	9.22	3.4
Polje	156	30–36 w in utero	Modern	Ossicle	−20.59	10.76	3.3
Polje	213	40 w in utero ±2 w	Modern	i1	−19.28	12.85	3.4
Polje	213	40 w in utero ±2 w	Modern	m1	−19.52	12.72	3.5
Polje	213	40 w in utero ±2 w	Modern	Rib	−19.5	10.79	3.4
Polje	213	40 w in utero ±2 w	Modern	Femur	−19.59	10.81	3.4
Polje	209	40 w in utero ±2 w	Modern	i1	−19.54	12.46	3.3
Polje	209	40 w in utero ±2 w	Modern	Rib	−19.36	10.94	3.4
Polje	209	40 w in utero ±2 w	Modern	Femur	−20.21	10.62	3.2
Polje	93	38 w in utero ±1 m	Modern	i1	−20.52	11.12	3.6
Polje	93	38 w in utero ±1 m	Modern	Rib	−19.44	10.53	3.4
Polje	93	38 w in utero ±1 m	Modern	Humerus	−19.58	10.39	3.4
Polje	196	36–38 w in utero	Modern	i1	−19.44	12.76	3.5
Polje	196	36–38 w in utero	Modern	Rib	−18.44	11.36	3.6
Polje	196	36–38 w in utero	Modern	Femur	−18.73	11.27	3.4
Polje	196	36–38 w in utero	Modern	Ossicle	−20.33	11.98	3.4
Polje	212	40 w in utero ±2 w	Modern	i1	−19.8	13	3.3
Polje	212	40 w in utero ±2 w	Modern	m2	−19.86	13.56	3.4
Polje	212	40 w in utero ±2 w	Modern	Rib	−19.27	11.36	3.3
Polje	212	40 w in utero ±2 w	Modern	Humerus	−19.42	11.3	3.3
Polje	135	1.5–4.5 m	Modern	m1	−18.96	10.79	3.3
Polje	135	1.5–4.5 m	Modern	Rib	−19.21	9.2	3.1
Polje	135	1.5–4.5 m	Modern	Humerus	−20.19	10.08	3.3
Polje	135	1.5–4.5 m	Modern	Ossicle	−21.52	10.78	3.4
Polje	107	1.5 ± 3 m	Modern	m2	−19.25	13.19	3.3
Polje	107	1.5 ± 3 m	Modern	Ossicle	−20.66	12.11	3.4
Polje	107	1.5 ± 3 m	Modern	Rib	−19.13	11.49	3.3
Polje	107	1.5 ± 3 m	Modern	Humerus	−19.13	11.36	3.2
Polje	8	1.5 ± 3 m	Modern	m2	−20.18	12.83	3.4
Polje	8	1.5 ± 3 m	Modern	Rib	−19.86	11.47	3.3
Polje	8	1.5 ± 3 m	Modern	Humerus	−19.47	11.93	3.3
Polje	8	1.5 ± 3 m	Modern	Ossicle	−21.29	13.18	3.3
Polje	138	1.5 ± 3 m	Modern	i1	−19.15	12.67	3.3
Polje	138	1.5 ± 3 m	Modern	m2	−19.36	12.81	3.4
Polje	138	1.5 ± 3 m	Modern	Femur	−19.31	12.09	3.4
Polje	138	1.5 ± 3 m	Modern	Ossicle	−19.99	11.41	3.3
Polje	5	1.5 ± 3 m	Modern	m1	−19.01	12.52	3.3
Polje	5	1.5 ± 3 m	Modern	Rib	−18.94	11.63	3.5
Polje	5	1.5 ± 3 m	Modern	Femur	−18.58	11.62	3.4
Polje	5	1.5 ± 3 m	Modern	Ossicle	−20.06	11.65	3.3
Polje	101	1.5 ± 3 m	Modern	i1	−19.27	12.49	3.2
Polje	101	1.5 ± 3 m	Modern	m2	−19.42	12.11	3.2
Polje	101	1.5 ± 3 m	Modern	Rib	−19.87	10.37	3.3
Polje	101	1.5 ± 3 m	Modern	Femur	−20	10.07	3.2

Abbreviations: m, months; w, weeks.

A total of 21 individuals who died in utero or shortly after birth (peri/neonates) and 6 individuals aged between approximately 1.5 and 4.5 months (infants) were included. All individuals were represented by at least one tooth (deciduous incisor or molar) and one bone (rib and/or long bone in one case a metacarpal was analyzed and treated within the long bone category), eight individuals also had ossicles (incus or malleus). Ribs and long bones were considered together because both develop through endochondral ossification and undergo continuous remodeling during late gestation, thereby representing comparable developmental stages. Although anatomically smaller than the major limb long bones, metacarpal develops through the same endochondral process and was therefore included in the long bone category. Similarly, deciduous incisors and molars were grouped as dental tissues, as both initiate mineralization during the second trimester and record overlapping prenatal isotopic signals.

For comparative analyses, individuals were grouped into three age categories based on osteological and dental development: perinate, neonate, and infant

- The perinate category (*n* = 13) includes individuals estimated to have died between approximately 30 and 40 weeks of gestation.
- The neonate group (*n* = 8) comprises individuals estimated to have died around the time of birth (40 weeks ±2 weeks).
- The infant group (*n* = 6) includes individuals who survived beyond the neonatal period, with estimated ages ranging from 1.5 to 4.5 months postnatal.

These age categories were assigned based on macroscopic assessment of skeletal development, primarily using crown formation and eruption stages of deciduous teeth, as well as diaphyseal lengths of long bones (AlQahtani et al. 2010; Scheuer and Black 2000). Age estimates derived from dental development and long bone length were then compared to identify potential discrepancies that might indicate growth stunting, often linked to chronic malnutrition or systemic stress (Lewis 2007). Because both approaches yield age ranges, a point estimate was calculated for each method as the midpoint of the reported range, and the dental–skeletal offset was expressed as *Δ* age = (dental midpoint) − (skeletal midpoint). Individuals were classified as stunted when the midpoint dental‐age estimate exceeded the midpoint long‐bone estimate by ≥ 4 weeks. To assess whether stunting was associated with variation in isotopic offsets among different skeletal tissues, individuals with observed discrepancies in dental development and long bone length were compared. *Stunted* individuals were defined as those displaying discrepancies between dental development and long bone length, indicating impaired growth. All other individuals, whose dental and skeletal maturation were concordant for age, were considered the *non‐stunted* comparison group.

The Polje site represents a 17th–19th century cemetery associated with the Modern period urban settlement in Ljubljana. Because burials include several well‐preserved perinatal, neonatal, and also infant individuals, this site served as the basis for within‐site comparisons of developmental isotopic variation.

### 2.2 Carbon and Nitrogen Isotope Analysis

Sample selection was based on the preservation quality of the remains; however, the inclusion criteria required that each individual contributed at least one deciduous tooth and one long bone or rib (see Table 1 for sample list). Altogether 106 samples were collected for the analysis. Each sampled element (tooth, ossicle, long bone, and rib) was initially cleaned using an ultrasonic bath.

Collagen extraction followed a modified Longin method (Brown et al. 1988; Longin 1971). Samples were demineralized in 0.5 M hydrochloric acid (HCl) at 4°C–5°C, with the acid replaced every few days until demineralization was complete. Both bones and teeth were processed using the same extraction protocol. Demineralized samples were rinsed with deionized water, then gelatinized in pH 3 HCl at 70°C for 24 to 48 h, or until the collagen was fully solubilized. The resulting solution was frozen and lyophilized (freeze‐dried), and the purified collagen was used for isotope measurement.

Isotope ratios of ^13^C/^12^C and ^15^N/^14^N were determined via Isotope Ratio Mass Spectrometry (IRMS) using the IsoPrime100—Vario PYRO Cube system. Approximately 0.5 mg of collagen sample was weighed into a tin capsule. The capsule was then closed with tweezers and put into the automatic sampler of the elemental analyzer. The results were normalized against the following international reference materials: IAEA‐600 with *δ*13C = −27.7‰ ± 0.0‰ and *δ*15N = +1.0 ± 0.1‰, USGS88 with *δ*13C = −16.1‰ ± 0.1‰ and *δ*15N = +15.0‰ ± 0.1‰, and USGS89 with *δ*13C = −18.1‰ ± 0.1‰ and *δ*15N = 6.3‰ ± 0.1‰. For the independent control laboratory reference material CRP‐IAEA casein with *δ*13C = −20.3‰ ± 0.1‰ and *δ*15N = +5.6‰ ± 0.2‰ were used. The analytical precision was ±0.2‰ (1SD) for both *δ* ^13^C and *δ* ^15^N values.

Five samples with a C:N atomic ratio below 2.9 or above 3.6, % C below 13%, and % N below 4.8% were excluded from further analysis, following widely accepted collagen quality criteria (Ambrose 1990; DeNiro 1985; van Klinken 1999). These thresholds are used to ensure that the isotopic values reflect endogenous collagen and are not significantly altered by diagenesis.

### 2.3 Statistical Analysis

All statistical analyses and visualizations were performed using RStudio (R 2025.05.0 Build 496). To evaluate differences in isotopic values between groups, non‐parametric statistical tests were employed due to small sample sizes and potential deviations from normality. The Wilcoxon rank‐sum test (Mann–Whitney *U* test) was used for comparisons involving two independent groups, such as *δ* ^13^C and *δ* ^15^N values between the T1 (first deciduous incisor or first deciduous molar) and T2 (second deciduous incisor or second deciduous molar) tooth types, between infants and merged peri/neonate groups, and between the individuals with and without the observed discrepancies in dental development and long bone length. In cases involving three or more groups, such as comparisons of isotopic values across the age group categories (perinate, neonate, infant), the Kruskal–Wallis test was applied. When significant differences were detected with Kruskal–Wallis, post hoc pairwise Wilcoxon tests were conducted to identify specific group differences. Despite the presence of outliers, all individuals were retained in the statistical analyses.

Because isotopic data often show considerable individual‐level variation, all individuals were also visually inspected for element‐by‐element trajectories. This step did not involve any prior expectation of outliers but allowed unexpected patterns to be identified and reported transparently.

## 3 Results

### 3.1 Osteological Analysis

Result of osteological analyses is summarized in Table 1. Thirteen individuals were assessed as perinates, eight as neonates and six as infants. In most individuals, dental development and long bone length provided consistent age estimates. However, in four infants from Polje (graves 8, 101, 107, 135), dental development suggested an age of approximately 1.5 months, whereas diaphyseal lengths were more consistent with in utero development (36–40 weeks), potentially indicating growth stunting or delayed postnatal development. In these cases, age from dental development was used since tooth formation is relatively buffered against short‐term physiological/environmental stress and shows less variability than skeletal maturation (Cardoso 2007; Conceição and Cardoso 2011).

### 3.2 Carbon and Nitrogen Isotope Analysis

**FIGURE 1:**
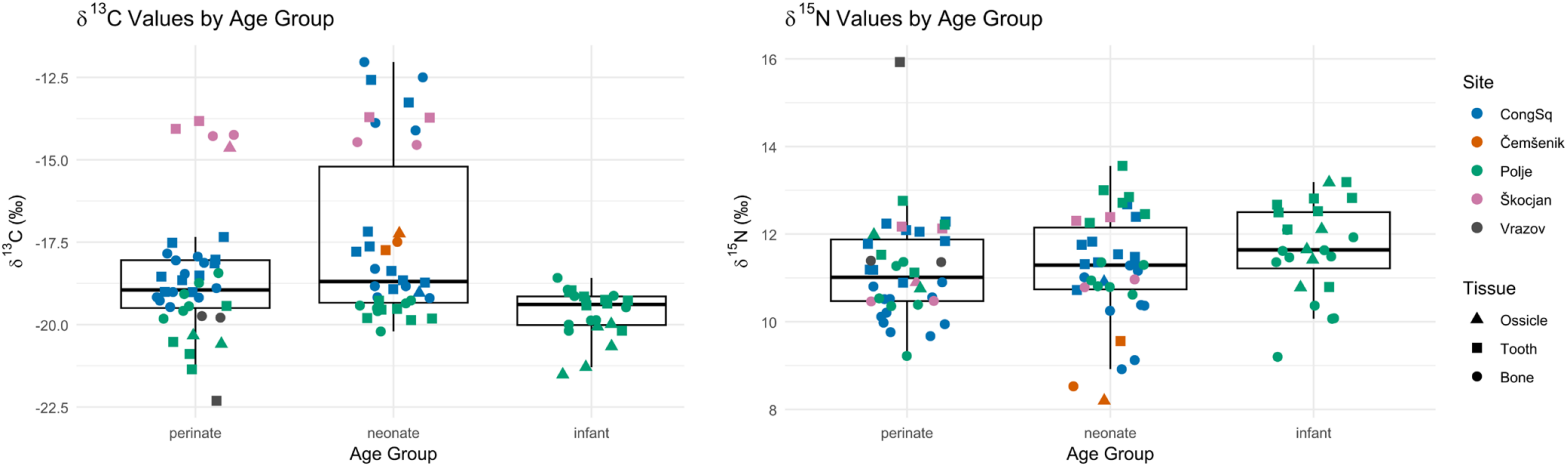
*δ* ^13^C and *δ* ^15^N values by age group (boxplot), site (color), and tissue (shape).

Results of isotope analyses are provided in Table 1 and Figure 1.

### 3.3 Comparisons of Isotopic Values Between Perinates, Neonates and Infants From Polje

**FIGURE 2:**
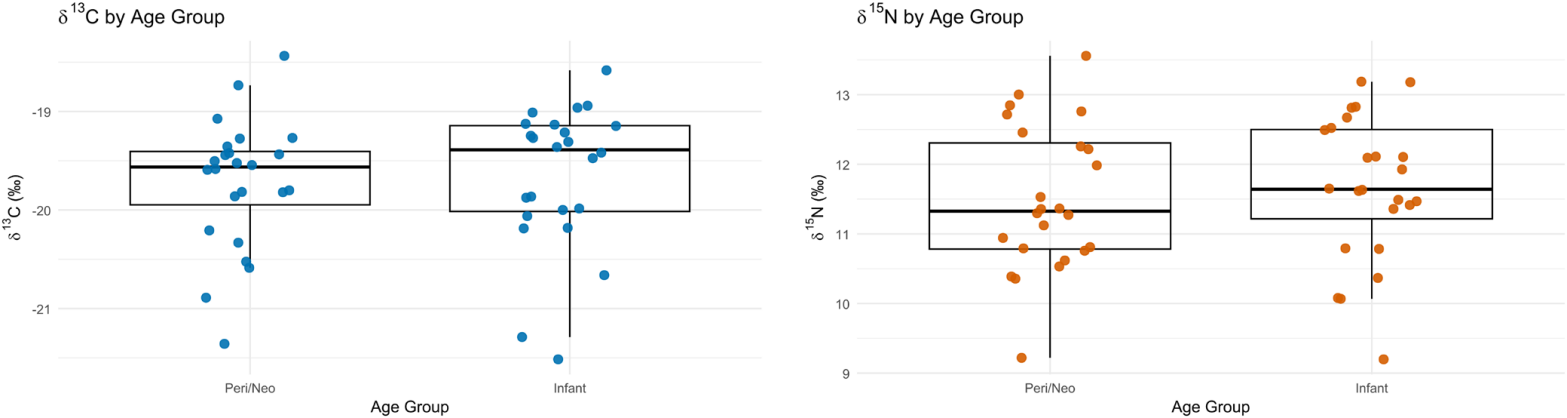
Boxplots comparing *δ* ^13^C and *δ* ^15^N values between a combined group of perinates and neonates and infants from the Polje site. The plots illustrate the distribution of carbon and nitrogen isotope values across age groups.

Polje was the only site in the study that included infant individuals. To assess whether significant isotopic differences exist between those who died in utero or around birth and those who survived into early infancy, infants from Polje were compared to the combined group of perinates and neonates from the same site (Figure 2). No statistically significant differences were observed in *δ* ^13^C or *δ* ^15^N values between perinates/neonates and infants from Polje (*δ* ^13^C: *W* = 329, *p* = 0.407; *δ* ^15^N: *W* = 325, *p* = 0.455) (Figure 2).

### 3.4 Comparison of Isotopic Values Between Long Bone and Rib

**FIGURE 3:**
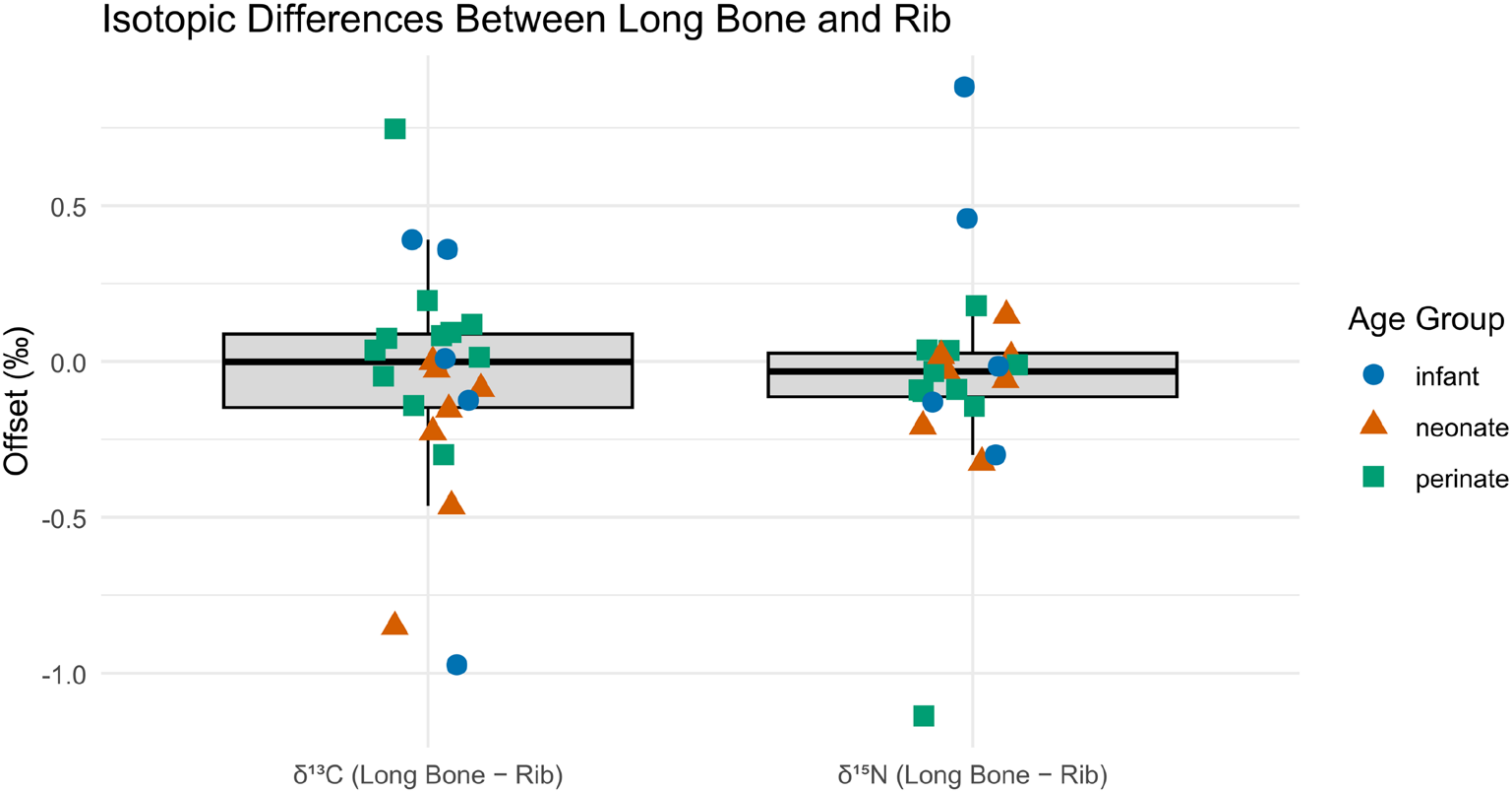
Isotopic differences between long bones and ribs for *δ* ^13^C and *δ* ^15^N values. Boxplots show the distribution of offsets (long bone minus rib) for each isotope, with overlaid individual data points. Data points are color‐ and shape‐coded by age group: Infants (blue circles), neonates (orange triangles), and perinates (green squares).

To assess the comparability of isotopic values between different skeletal elements (Figure 3), systematic offsets between long bone and rib collagen within individuals (*n* = 23) were tested. Results indicate no significant difference in either *δ* ^13^C or *δ* ^15^N values between long bone and rib (*δ* ^13^C: *V* = 119, *p* = 0.574; *δ* ^15^N: *V* = 97, *p* = 0.218). Based on these results, *δ* ^13^C and *δ* ^15^N values from ribs and long bones were either averaged or used interchangeably in subsequent analyses.

### 3.5 Comparison of Isotopic Values Between T1 and T2

**FIGURE 4:**
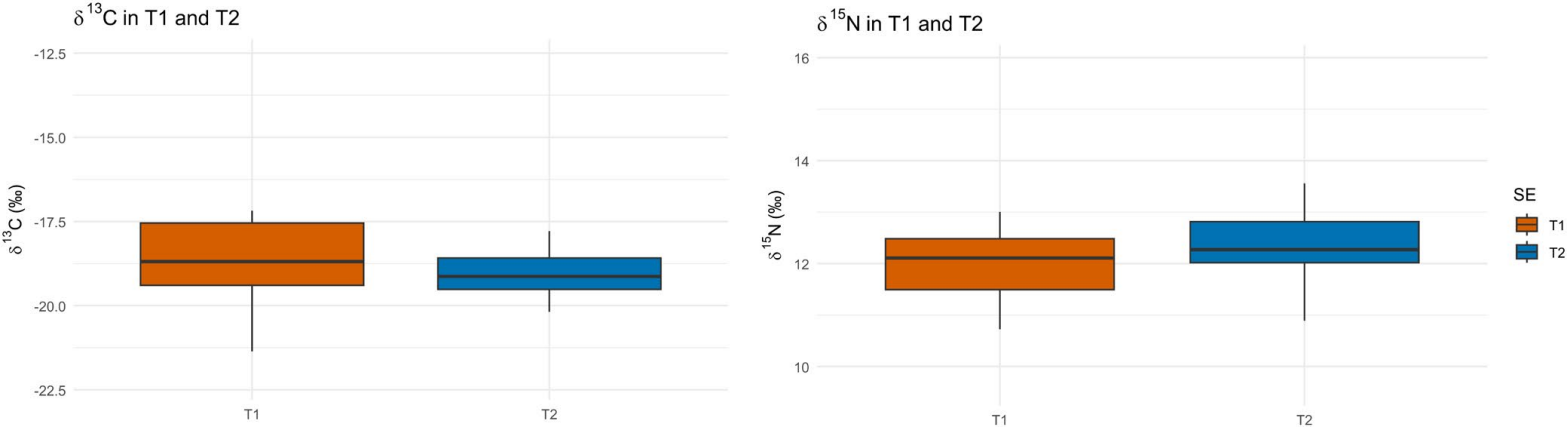
Boxplots comparing *δ* ^13^C and *δ* ^15^N values between two tooth formation phases: T1 (first incisor and first molar) and T2 (second incisor and second molar).

To assess the consistency of isotopic signals within the dentition, *δ* ^13^C and *δ* ^15^N values were compared between two deciduous teeth sampled from the same individuals (first incisor and first molar versus second incisor and second molar; *n* = 6) (Figure 4). As the teeth represent different formation periods, this comparison allows for the evaluation of intra‐individual dietary or metabolic variation during early development.

The Wilcoxon rank‐sum test indicated no statistically significant differences in either isotope ratios (*δ* ^13^C: *W* = 218, *p* = 0.296; *δ* ^15^N: *W* = 137, *p* = 0.237). These results suggest that, within the sampled individuals, T1 and T2 dentin preserve comparable isotopic signals, indicating that dentin collagen from different deciduous teeth formed in early life reliably reflects consistent dietary and/or metabolic signals. Based on these results, *δ* ^13^C and *δ* ^15^N values from T1 and T2 teeth were either averaged or used interchangeably in subsequent analyses.

### 3.6 Comparison of Isotopic Values Between Tooth and Long Bone/Rib

**FIGURE 5:**
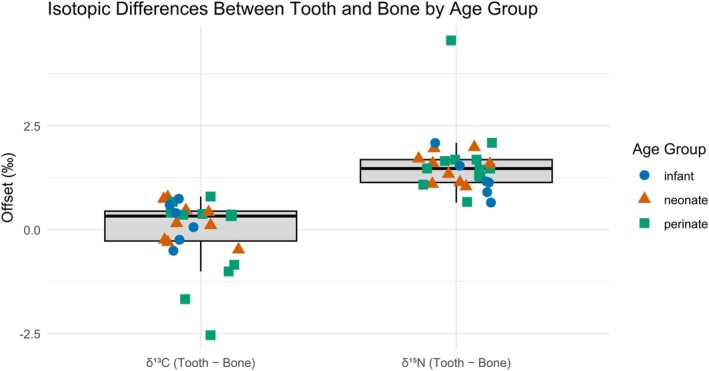
Isotopic differences between teeth and long bones/ribs for *δ* ^13^C and *δ* ^15^N values, grouped by age category. Boxplots represent the distribution of offsets (tooth minus bone) for each isotope, with individual data points overlaid and color/shape‐coded by age group: Infants (blue circles), neonates (orange triangles), and perinates (green squares).

To evaluate differences in *δ* ^13^C and *δ* ^15^N values between tooth and long bone/rib tissues (Figure 5), Wilcoxon signed‐rank tests were conducted both across the full sample and within each age group (perinate, neonate, infant). Across all individuals, no statistically significant offset was observed for *δ* ^13^C (*V* = 228, *p* = 0.355). However, the relatively high variability in carbon values may obscure subtle tissue‐specific differences. Therefore, while tooth and long bone/rib *δ* ^13^C values appear broadly similar at the group level, this may reflect inconsistent patterns rather than true equivalence. In contrast, a highly significant positive offset was found for *δ* ^15^N (*V* = 378, *p* < 0.00001), indicating that *δ* ^15^N values were consistently higher by 1.5‰ on average in tooth tissue compared to long bone/rib.

The age‐specific tests for *δ* ^13^C revealed no significant differences in any age group (Infant: *p* = 0.402, *W* = 15; Neonate: *p* = 0.343, *W* = 31; Perinate: *p* = 0.845, *W* = 36). In contrast, *δ* ^15^N showed a significant offset in all three age groups (Infant: *p* = 0.036, *W* = 21; Neonate: *p* = 0.00915, *W* = 45; Perinate: *p* = 0.00253, *W* = 78).

### 3.7 Comparison of Isotopic Values Between Ossicle and Long Bone/Rib and Teeth

**FIGURE 6:**
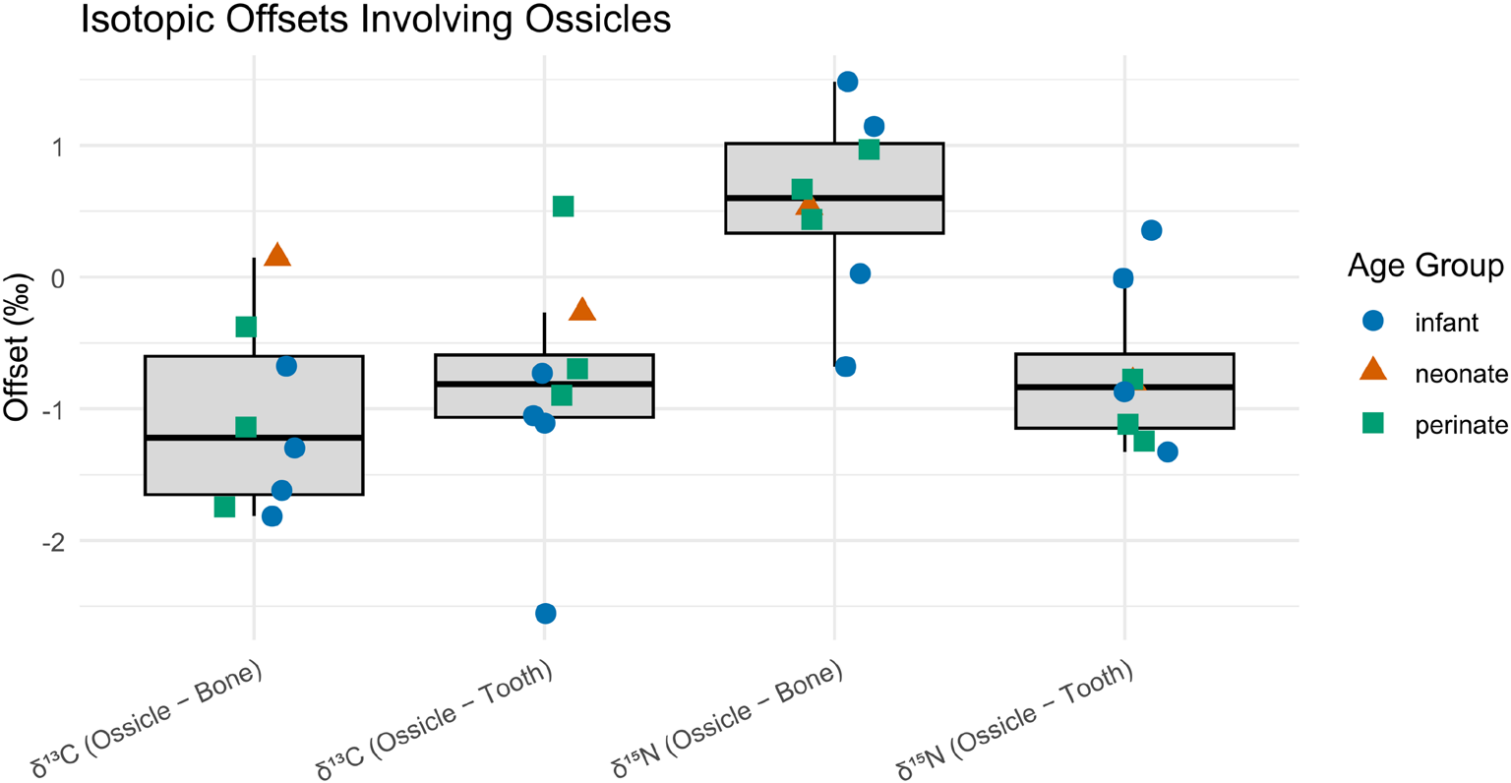
Isotopic offsets involving ossicles compared to long bone/rib and tooth values for both *δ* ^13^C and *δ* ^15^N values. Boxplots display the distribution of isotopic differences (ossicle minus long bone/rib or tooth), with individual data points overlaid and color/shape‐coded by age group: Infants (blue circles), neonates (orange triangles), and perinates (green squares).

To explore isotopic differences involving ossicles, *δ* ^13^C and *δ* ^15^N values between ossicles, long bone/rib, and teeth were compared for the eight individuals for which an ossicle was available (Figure 6). Across all individuals, ossicles showed a significantly lower *δ* ^13^C value compared to both long bone/rib (*V* = 1, *p* = 0.021) and tooth (*V* = 2, *p* = 0.030). This consistent negative offset in *δ* ^13^C is clearly visible in the plot, with most ossicle values falling below both skeletal elements. In contrast, *δ* ^15^N offsets were more variable. While ossicle values were significantly lower than teeth (*V* = 2, *p* = 0.030), they showed a non‐significant but positive trend compared to long bone/rib (*V* = 31, *p* = 0.080), suggesting that ossicles may incorporate dietary nitrogen differently. Although age groups (infant, neonate, perinate) were included in the visualization, statistical comparisons by age were not conducted due to the limited sample size–only 8 individuals had ossicles. Interestingly, the plot suggests that infants stand out more in terms of *δ* ^15^N offsets, potentially indicating postnatal dietary influence or physiological changes (e.g., stress) after birth.

### 3.8 Comparison of Isotopic Tissue to Tissue Offsets Between Stunted and Non‐Stunted Individuals

**FIGURE 7:**
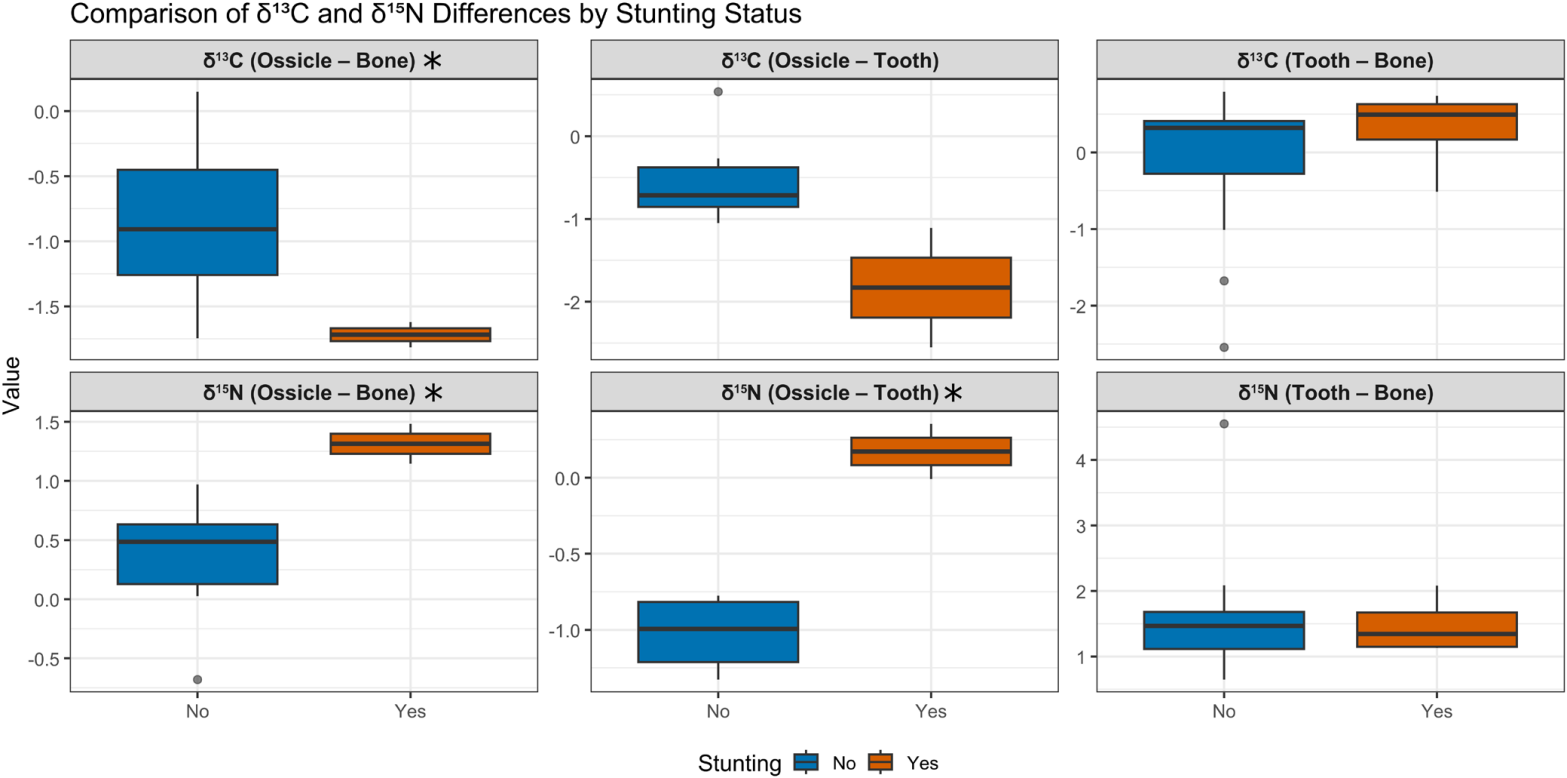
Boxplots showing *δ* ^13^C and *δ* ^15^N isotopic offsets (‰) between bone, tooth, and ossicle tissues for stunted (Yes) and non‐stunted (No) individuals. Offsets represent the difference in *δ*‐values between paired tissues: *δ* ^13^C (Tooth–Bone), *δ* ^15^N (Tooth–Bone), *δ* ^13^C (Ossicle–Bone), *δ* ^15^N (Ossicle–Bone), *δ* ^13^C (Ossicle–Tooth), and *δ* ^15^N (Ossicle–Tooth). Significant differences between stunting groups (*p* < 0.05) are indicated by an asterisk.

Significant differences in isotopic offsets between stunted (Polje graves 8, 101, 107, 135) and non‐stunted individuals were observed primarily in tissue comparisons involving ossicles (Figure 7). Stunted individuals exhibited a significantly larger negative *δ* ^13^C (Ossicle–Bone) offset compared with non‐stunted individuals (*p* = 0.0267), indicating that ossicle *δ* ^13^C values were more depleted relative to bone. Stunted individuals also showed significantly higher *δ* ^15^N (Ossicle–Bone) offset compared with non‐stunted (*p* = 0.0194), reflecting a substantially increased *δ* ^15^N enrichment in ossicles relative to bone. A similar pattern was apparent for *δ* ^15^N (Ossicle–Tooth), which was also significantly higher in the stunted individuals (*p* = 0.0443). No significant differences were detected for *δ* ^13^C (Ossicle–Tooth), *δ* ^13^C (Tooth–Bone), or *δ* ^15^N (Tooth–Bone) offsets (*p* > 0.05). Overall, isotopic offsets involving ossicle tissue consistently distinguished stunted and non‐stunted individuals.

## 4 Disscussion

### 4.1 General Interpretation

**FIGURE 8:**
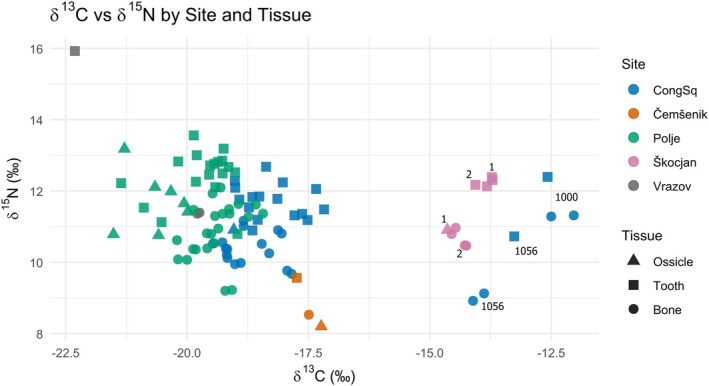
Distribution of *δ* ^13^C and *δ* ^15^N values for all individual samples included in this study. Each point represents a single measurement, grouped by site and tissue type.

Clear differences in *δ* ^13^C values are evident among several individuals, notably those from Škocjan (Grave 1 and 2, plotted in blue) and two neonates from Congress Square (Graves 1000 and 1056) (Figure 8). These individuals exhibit significantly less negative *δ* ^13^C values than the rest of the sample, suggesting a greater input of C_4_‐derived foods in the maternal diet (Katzenberg 2008; Schwarcz and Schoeninger 1991). This interpretation is particularly compelling for Graves 1000 and 1056 from Congress Square, as they differ markedly from other neonates buried at the same site, all of whom display more negative *δ* ^13^C values consistent with a C_3_‐based terrestrial diet. Importantly, this pattern does not appear to result from marine resource consumption, as *δ* ^15^N values in these individuals are not elevated (Schoeninger et al. 1983; Schoeninger and DeNiro 1984). In fact, Grave 1056 from Congress Square shows one of the lowest *δ* ^15^N values in the entire dataset. This case is of particular interest, as Grave 1056 is the only peri/neonate from Congress Square dated to the Early Iron Age, whereas the remaining individuals from this site are associated with Early Roman contexts. Similarly, the two Škocjan individuals with higher *δ* ^13^C values are dated to the Late Iron Age. These chronological distinctions strengthen the interpretation that the elevated *δ* ^13^C values may reflect broader dietary shifts over time, likely linked to increased consumption of C_4_ plants, such as millet, during the later prehistoric periods in contrast to the Roman diet. Archeobotanical and isotopic evidence indicates that millet became increasingly important in Iron Age Slovenia (Nicholls and Koon 2016; Tolar and Pavlin 2022), whereas Roman‐period Emona shows predominantly C_3_‐based diets with only limited C_4_ input, consistent with reduced millet consumption in the Roman era (Leskovar et al. 2025).

Additionally, some individuals display outlying *δ* ^15^N and *δ* ^13^C values, including those from Vrazov trg and Čemšenik. Detailed discussion of these outliers is provided in Section 4.5.

### 4.2 Peri/Neonates vs. Infants

Although the infants were estimated to be approximately 1.5 months old (with one between 1.5 and 4.5 months), they do not exhibit the elevated *δ* ^13^C or *δ* ^15^N values typically associated with breastfeeding (Fogel et al. 1989; Fuller et al. 2006). This absence of a breastfeeding signal, contrary to observations by Richards et al. (2002) and Prowse et al. (2008), who documented rapid incorporation of breast milk protein into infant bone collagen, could suggest that the isotope signal from breastmilk had not yet been incorporated into bone collagen due to the very short postnatal interval, during which prenatal collagen still dominates and/or that feeding was supplemented or partially replaced by alternative food sources, a practice well documented in 17th–19th century Europe (Fildes 2017; Stevens et al. 2009). Supporting this, four out of six infants display discrepancies between age estimates based on dental development and long bone length, consistent with stunted growth and possibly poor nutritional status. Furthermore, the oldest individual (Grave 135) shows a decrease in *δ* ^15^N from tooth to long bone/rib, which contradicts the typical breastfeeding trajectory and may indicate a premature cessation of exclusive breastfeeding or the introduction of supplementary foods or liquids earlier than expected.

It is also important to consider that some infants may have survived long enough to initiate breastfeeding and that higher *δ* ^15^N in dentin could, in part, reflect incorporation of breast milk proteins (Fogel et al. 1989; Fuller et al. 2006; Prowse et al. 2008; Richards et al. 2002). However, this explanation alone cannot account for the consistent offset between tissues because perinates, who most likely did not survive to be breastfed, show the same pattern of elevated *δ* ^15^N in dentin relative to ossicles and long bone/rib. Thus, while early breastfeeding could accentuate the *δ* ^15^N signal in some infants, the presence of the same offset in individuals unlikely to have breastfed indicates that tissue‐specific collagen formation and in utero physiological conditions are the primary drivers of this pattern.

### 4.3 Stunted and Non‐Stunted Individuals

In this study, stunted individuals were defined as those displaying discrepancies between dental development and long bone length, indicating impaired growth. All other individuals, whose dental and skeletal maturation were concordant for age, were considered the non‐stunted comparison group. The isotopic differences between these groups can be explained by how early‐life physiological stress influences collagen synthesis and how different tissues record metabolic conditions during fetal and neonatal development. Stunting typically reflects chronic undernutrition, recurrent infection, and reduced growth velocity, all of which elevate *δ* ^15^N through increased nitrogen recycling and catabolic activity and can shift *δ* ^13^C via altered carbon routing (Fuller et al. 2005a; Beaumont et al. 2015; Richards et al. 2002). Although tooth–bone isotopic offsets exist at the sample set level, these differences are systematic and reflect normal developmental biology. Tooth dentin consistently shows higher *δ* ^15^N values than bone collagen across all individuals, due to its fixed formation window and lack of remodeling (AlQahtani et al. 2010; Hillson 2014). When comparing stunted and non‐stunted individuals, the magnitude of the tooth–bone offset remains essentially identical, demonstrating that this spacing is not sensitive to growth impairment. Offsets involving ossicles therefore show the clearest separation between stunted and non‐stunted individuals. The more negative *δ* ^13^C (Ossicle–Bone) offsets and the markedly elevated *δ* ^15^N (Ossicle–Bone) and *δ* ^15^N (Ossicle–Tooth) offsets in stunted individuals indicate patterns consistent with disrupted fetal growth and heightened metabolic stress (Beaumont et al. 2015; Mekota et al. 2006). The fact that only ossicle‐involved offsets differentiate the groups suggests that the metabolic stress associated with growth impairment occurred primarily during mid‐ to early late gestation, when ossicles were forming most intensively. Tooth dentin, which begins forming later (~30 gestational weeks) and continues into early infancy, shows no such distinction, indicating that physiological conditions during its formation window were either more similar across individuals or less extreme than during ossicle formation. Bone collagen, which remodels rapidly, further smooths early fetal signals. Together, these patterns imply that the stress underlying stunting was most pronounced earlier in fetal development and was no longer differential between groups during late gestation and early postnatal life. It should be noted, however, that the number of individuals available for isotopic comparison is limited, and therefore the interpretations presented here remain provisional and require validation in larger datasets.

### 4.4 *δ* ^15^N and *δ* ^13^C Values Across Tissues and Their Implications

As all analyzed individuals died in utero, at birth, or shortly thereafter, these isotopic signals reflect only those who did not survive infancy and should be interpreted within the limitations of the osteological paradox (Wood et al. 1992). Moreover, the individuals analyzed in this study are exclusively perinates, neonates, and very young infants. While auditory ossicles are understood to complete ossification prenatally and undergo only minimal postnatal remodeling, our data cannot determine whether ossicle collagen values remain stable throughout later childhood or adulthood. The isotopic patterns documented here therefore apply specifically to early‐life tissues, and additional research on older individuals is required to assess whether ossicles retain a fixed prenatal signature or experience subtle remodeling later in life.

**FIGURE 9:**
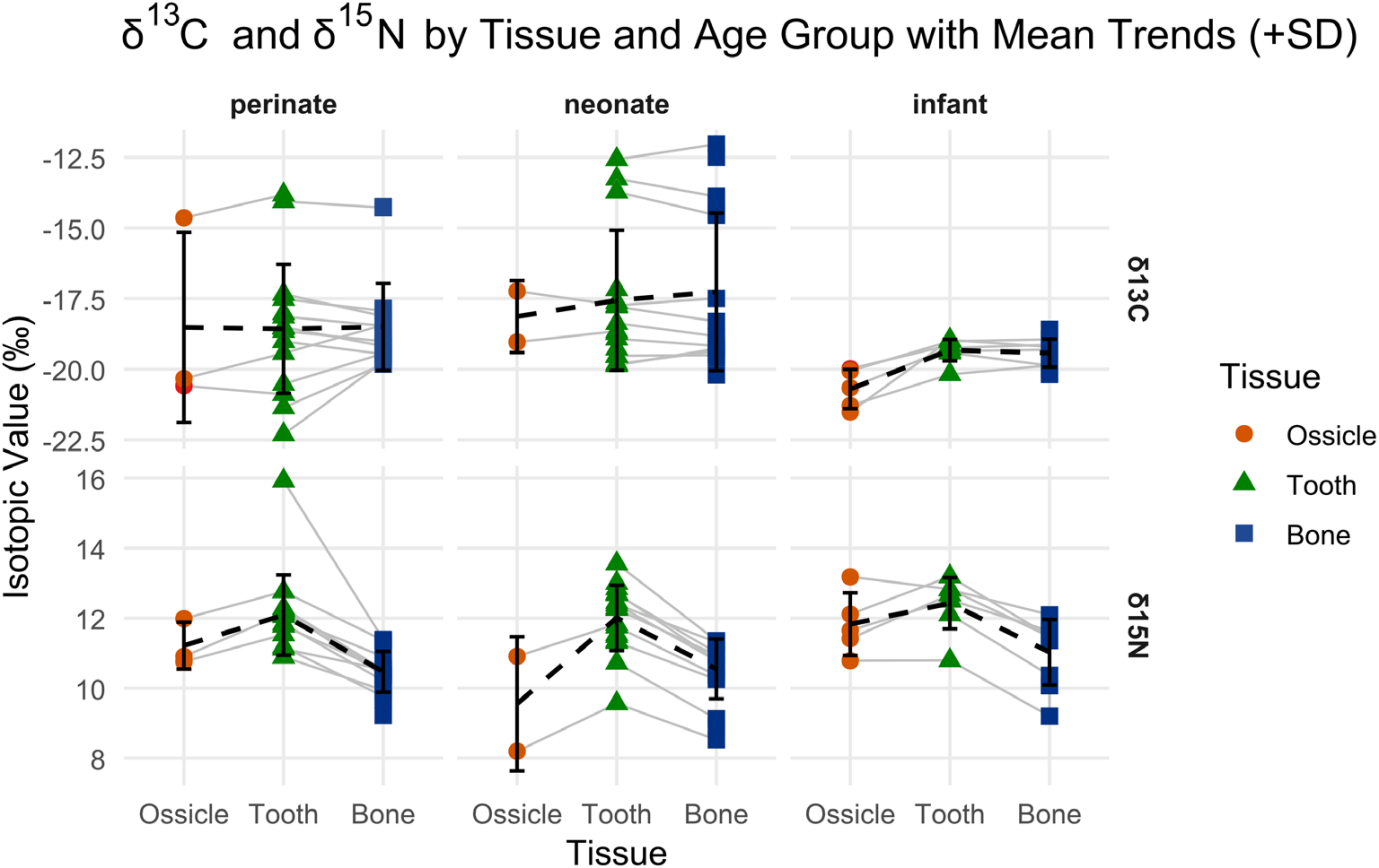
*δ* ^13^C and *δ* ^15^N values by tissue type (ossicle, tooth, long bone/rib) and age group (infant, neonate, perinate), with mean trends (+SD) overlaid. Each panel shows individual isotopic values (*δ* ^13^C in the top row, *δ* ^15^N in the bottom row) by tissue: Ossicle (red circles), tooth (green triangles), and long bone/rib (blue squares).

*δ* ^15^N values exhibit a consistent directional trend across all age groups, increasing from ossicle (forming predominantly during the second trimester) to tooth (forming predominantly during the third trimester) and subsequently decreasing from tooth to long bone/rib (forming over a broader time frame with continuous modeling and remodeling). This pattern is clearly visible in both individual‐level trajectories and group‐level means (Figures 8 and 9). These differences can hardly be attributed to dietary changes, as most individuals analyzed died in utero or shortly after birth. Attributing the pattern to maternal diet implies a synchronous and homogeneous shift across mothers from five separate locations and four different periods, an inference incompatible with the archeological independence and temporal dispersion of the sample.

Acute or chronic stress (e.g., infection, starvation, metabolic disorders) can elevate *δ* ^15^N via catabolism and may contribute to some offsets (Beaumont et al. 2015; Beaumont and Montgomery 2016; Fuller et al. 2006; Mekota et al. 2006; Reitsema 2013). This interpretation is supported by work showing that physiological stress and associated tissue catabolism can generate intra‐skeletal isotope differences. Curto et al. (2020) demonstrated systematic *δ* ^13^C and *δ* ^15^N differences between lesioned and non‐lesioned skeletal tissues within individuals. Such findings underscore that stress‐related remodeling and metabolic routing can modulate collagen isotope ratios and must be considered when evaluating tissue‐to‐tissue offsets. However, three features argue against stress as the primary driver of the systematic pattern observed here: (i) the consistent directional trend (ossicle < tooth > long bone/rib) across age groups, including perinates unlikely to have experienced prolonged extra‐uterine catabolism; (ii) the absence of uniformly elevated *δ* ^15^N across tissues and individuals, with both high (e.g., Vrazov trg) and low (e.g., Čemšenik) outliers, suggesting heterogeneous stress histories; and (iii) the magnitude and tissue order of offsets align with expectations from formation timing, remodeling, and metabolic routing rather than generalized stress effects (Beaumont and Montgomery 2016; Reitsema 2013). Therefore, stress is a possible modifier in specific cases but cannot be the dominant explanation for this cross‐tissue pattern.

Higher *δ* ^15^N values in ossicle compared to tooth dentin may relate to increasing maternal‐fetal nitrogen transfer efficiency later in gestation, which is known to result in isotopic fractionation that enriches fetal tissues in ^15^N (Fuller et al. 2006; Tsutaya and Yoneda 2015). Ossicles, which ossify early in gestation (second trimesters), are likely formed when placental nitrogen transfer is still relatively inefficient, resulting in lower *δ* ^15^N values. In contrast, deciduous tooth dentin forms predominantly during the third trimester, a period marked by rapid fetal growth and heightened amino acid transfer, leading to greater ^15^N enrichment (or higher *δ* ^15^N values). This pattern aligns with evidence that nitrogen is primarily transferred from mother to fetus as amino acids, and that isotopic fractionation during this process enriches fetal tissues in ^15^N (Fuller et al. 2006; Herring et al. 1998). Following this, the decrease in *δ* ^15^N values from tooth to long bone/rib likely reflects a combination of physiological and biochemical mechanisms. Bone collagen in long bones and ribs, even in perinatal individuals, forms over a broader time frame and undergoes continuous modeling and remodeling. As a result, it may integrate isotopic signals across different developmental stages, effectively dampening the higher *δ* ^15^N values observed in more time‐restricted tissues like dentin (Beaumont et al. 2018; Katzenberg and Lovell 1999; Reitsema 2013). In addition, tissue‐specific metabolic routing of nitrogenous amino acids, especially those enriched in ^15^N, such as glutamine and glutamate, may contribute to higher *δ* ^15^N values in tooth dentin relative to long bone/rib (Froehle et al. 2010; O'Connell et al. 2012).

Taken together, the *δ* ^15^N differences observed between ossicle, tooth, and long bone/rib collagen in studied individuals are best explained by a combination of tissue‐specific formation timing, remodeling rates, and metabolic routing effects. These findings align with previous observations that even tissues forming simultaneously may differ isotopically due to physiological and biochemical mechanisms beyond simple dietary input (Beaumont et al. 2018).

In contrast to the more pronounced variation in *δ* ^15^N values, *δ* ^13^C values display a subtler yet consistent increase from ossicle to tooth across all age groups (perinates, neonates, and infants) (Figure 9). This directional trend, while modest in magnitude, is evident in the group means and suggests systematic differences in carbon incorporation during fetal development. The absence of a consistent shift from tooth to long bone/rib *δ* ^13^C values across individuals points to a relative isotopic convergence in late‐forming tissues, likely reflecting shared maternal dietary inputs during the third trimester and early postnatal life.

The observed increase in *δ* ^13^C values from ossicle to tooth may reflect a combination of physiological and temporal factors. Ossicles begin ossifying early in the second trimester and continue developing into the second and early third trimester, while deciduous tooth dentin forms primarily during the third trimester. The small yet consistent increase in *δ* ^13^C values from ossicle to tooth likely reflects not only this developmental timing but also maternal metabolic changes in late gestation, particularly shifts in carbohydrate and lipid metabolism, rather than differences in maternal diet alone (Beaumont et al. 2018; Beaumont and Montgomery 2016; Reitsema 2013). These subtle isotopic shifts are consistent with the influence of maternal physiology on fetal tissue composition via metabolic routing during the final stages of pregnancy.

Additionally, tissue‐specific differences in collagen biosynthesis and turnover may contribute to these isotopic offsets. Ossicles undergo limited remodeling, while dentin does not remodel at all, preserving a temporally constrained isotopic signal. Differences in amino acid routing and protein synthesis pathways between early‐ and later‐forming tissues may lead to modest yet consistent higher *δ* ^13^C values in dentin relative to ossicles (Fernandes et al. 2012; Fuller et al. 2005b). The absence of a systematic offset between tooth and long bone/rib *δ* ^13^C values may indicate that *δ* ^13^C values are more sensitive to inter‐individual variation in maternal diet and physiology than to developmental processes alone.

Taken together, the modest but consistent increase in *δ* ^13^C values from ossicle to tooth likely reflects a combination of gestational timing, maternal diet, and tissue‐specific metabolic routing. These findings reinforce the importance of considering tissue formation timing when interpreting *δ* ^13^C values in perinates, neonates, and young infants and caution against assuming isotopic equivalency across skeletal elements in such individuals.

### 4.5 Profiles of Individual (Outliers)

Given the relatively small number of individuals, outliers should be interpreted cautiously. The isotopic shifts observed in these cases are biologically plausible and consistent with known patterns of fetal and perinatal metabolic stress; however, individual‐level variation highlights that early‐life isotopic signatures can diverge sharply even within small cohorts. While these outliers do not contradict overall developmental trajectories identified in the sample, they underscore the importance of tissue‐aware sampling and careful interpretation when working with limited assemblages. Broader generalizations should therefore be made conservatively and ideally tested against larger comparative datasets.

**FIGURE 10:**
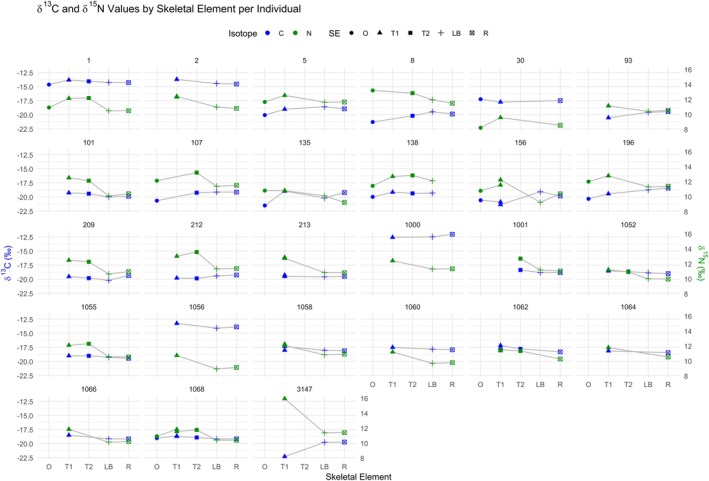
*δ* ^13^C (blue) and *δ* ^15^N (green) values plotted by skeletal element for each individual. Skeletal elements include ossicle (O), first‐forming teeth (T1), second‐forming teeth (T2), long bone (LB), and rib (R). Shapes distinguish element types, and isotope values are connected with lines to highlight intra‐individual trends.

Visual inspection (Figure 10) reveals several notable patterns and outliers: while most individuals show relatively stable values across tissues, some exhibit clear offsets. Variability in *δ* ^15^N values across elements may reflect developmental timing, tissue‐specific turnover, or physiological stress. *δ* ^13^C values are generally more stable but show distinct shifts in a few individuals.

Perinate 3147 from Vrazov trg presented a marked rise in *δ* ^15^N values from long bone and rib to tooth (4.6‰) and a sharp fall in *δ* ^13^C values from ossicle to tooth (2.6‰) (outlier; Figures 5 and 6). Pattern is consistent with in utero metabolic stress (e.g., maternal undernutrition) driving protein catabolism (Beaumont and Montgomery 2016).

Perinate 156 from Polje has lowest *δ* ^13^C and *δ* ^15^N at the site/age group: *δ* ^15^N values rise but *δ* ^13^C values fall from ossicle to tooth (outlier; Figure 6), pointing to physiological stress. Subsequent tooth to long bone offset shows *δ* ^15^N drop with modest *δ* ^13^C rebound (outlier; Figure 5), consistent with return toward baseline after a transient episode. Long bone to rib offsets (Figure 3) again shows opposing covariance, indicative of physiological stress (Beaumont and Montgomery 2016; Fuller et al. 2005b; Reitsema 2013).

Infant 135 from Polje presents lowest *δ* ^15^N values among Polje infants, and ossicle has the lowest *δ* ^13^C values. Unlike others, *δ* ^15^N values show no increase from ossicle to tooth, and *δ* ^13^C values rise more than typical by 2.6‰ (outlier; Figure 6). Minimal tooth to long bone change and a dental (1.5–4.5 months) versus skeletal (38–40 weeks in utero) age mismatch suggest suppressed postnatal isotopic shift (e.g., muted breastfeeding signal) or stress‐limited turnover. Long bone to rib shows decrease in *δ* ^15^N values and increase in *δ* ^13^C values (Figure 3), compatible with early dietary transition or physiological stress (Beaumont et al. 2013; Fuller et al. 2006; Tsutaya and Yoneda 2015).

Ossicle of infant 8 from Polje shows a high *δ* ^15^N value (outlier; Figures 1 and 6) with very low *δ* ^13^C value (inverse of most cases), pointing to early gestational stress (e.g., protein catabolism; lipid reliance) (Beaumont and Montgomery 2016; Fuller et al. 2005b; O'Connell et al. 2012). *δ* ^15^N values decline from ossicle to tooth, suggesting partial alleviation later in gestation; long bone to rib shows both isotopes decrease (Figure 3), consistent with dietary transition and/or stress reduction (Beaumont et al. 2013; Fuller et al. 2006; Tsutaya and Yoneda 2015).

## 5 Conclusions

This study provides the first systematic comparison of *δ* ^13^C and *δ* ^15^N collagen values across auditory ossicles, deciduous dentin, ribs, and long bones in perinatal, neonatal, and early infant individuals. Three clear isotopic patterns were identified across all sites: (1) *δ* ^15^N increases from ossicles to teeth and decreases again from teeth to long bone/rib; (2) *δ* ^13^C consistently rises from ossicle to tooth; and (3) no uniform *δ* ^13^C offset exists between tooth and long bone/rib. These findings demonstrate that isotopic variation in very young individuals is governed primarily by developmental biology—specifically tissue formation windows, nitrogen routing, and collagen turnover—rather than dietary differences.

A key outcome of this study is the identification of stable, predictable tissue‐based offsets, which provide a robust developmental framework for interpreting collagen isotopes in perinatal neonatal and early infant remains. Only ossicle–bone and ossicle–tooth offsets differentiate stunted from non‐stunted individuals, indicating that growth‐impairing stress was concentrated in mid‐ to late gestation and that tooth and bone do not retain these short‐lived fetal signals. The absence of a breastfeeding enrichment signal, even in infants up to 4.5 months old, further underscores the dominance of developmental and physiological processes over dietary signatures during this life stage.

Together, these results highlight the critical importance of tissue‐aware isotopic interpretation in studies of maternal diet, fetal physiology, and early‐life stress. They also demonstrate that mixing tissues across developmental windows can create misleading cross‐study comparisons. The offsets documented here establish essential baseline expectations for *δ* ^13^C and *δ* ^15^N trajectories in fetal and neonatal collagen and show how deviations from these patterns can serve as sensitive indicators of physiological stress. Future work incorporating incremental dentin sampling and modern comparative datasets will refine these developmental models and enhance our ability to reconstruct early‐life experiences.

## Author Contributions

**Tamara Leskovar:** conceptualization, investigation, writing – original draft, visualization, writing – review and editing. **Doris Potočnik:** investigation, methodology. **Marjeta Mencin:** formal analysis. **Nives Ogrinc:** funding acquisition, supervision. **Christophe Snoeck:** writing – review and editing, funding acquisition. **Matija Črešnar:** funding acquisition, writing – review and editing, supervision.

## Funding

This research was supported by the Research Foundation Flanders (FWO) and the Slovenian Research Agency (ARRS) through the FWO–ARRS Weave project CRIME (FWO G0A9721N; ARRS N7‐0194); the Research Infrastructure of the Slovenian Research and Innovation Agency (ARIS) (Archaeology, P6‐0247); and the MATRES Project, approved within the University of Ljubljana's preparatory scheme for large interdisciplinary projects and funded by ARIS (development pillar, RSF‐A).

## Ethics Statement

This research was conducted on human skeletal remains from archeological contexts in Slovenia. In accordance with Slovenian regulations, no additional institutional ethics approval was required for the analysis of ancient human remains. All procedures followed the ethical guidelines of the European Association of Archaeologists (EAA) and the International Council of Museums (ICOM) Code of Ethics for the treatment of archeological human remains.

## Conflicts of Interest

The authors declare no conflicts of interest.

## Supporting information


**Table S1:** Sample list with the results of osteological and isotope analyses.

## Data Availability

The raw data that supports the findings is included in the Supporting Information.
